# Serum albumin level predicts survival after surgical treatment of metastatic femur fractures: a retrospective study

**DOI:** 10.1186/s13018-020-01632-7

**Published:** 2020-04-03

**Authors:** David Shaoen Sim, Suraya Zainul-Abidin, Eileen Yilin Sim, Chu Sheng Seng, Shern-En Evan Tan, Mann Hong Tan, Tet Sen Howe, Joyce Suang Bee Koh

**Affiliations:** 1 Singapore General Hospital, Outram Rd, Singapore, 169608 Singapore; 2 Singapore, Singapore

**Keywords:** Albumin, Risk factor, Survival, Metastatic femur fracture

## Abstract

**Background:**

Surgical treatment for metastatic pathological femur fractures is associated with high mortality. Correct estimation of prognosis helps in determining the palliative value of surgical treatment and informs surgical decision. This study evaluates the risk factors for mortality in these patients who were surgically treated.

**Methods:**

This is a retrospective study of 112 patients with surgical treatment of metastatic pathological femur fractures. Risk factors evaluated included age, ASA status, Charlson comorbidity index, preoperative serum albumin and haemoglobin, primary tumour site, presence of visceral metastases, presence of spinal metastases, time from diagnosis of cancer to occurrence of pathological fracture, type of surgical procedure performed, lesion and whether treatment was received for an actual or impending fracture. A Cox regression model was used to determine if these factors were independent significant factors for survival.

**Results:**

Mortality at 2 years after surgical treatment of metastatic femoral fractures was 86%. Cox regression analysis of risk factors revealed that preoperative serum albumin and type primary tumour were independent risk factors for mortality. Presence of visceral metastases was strongly correlated to serum albumin levels.

**Conclusion:**

Preoperative serum albumin level and primary tumour site are independent risk factors of survival in patients treated for pathological femur fractures. Serum albumin level may be used as a prognostic tool to guide treatment in this cohort of patients with high mortality rates.

## Background

Longer survival times of cancer patients increase the risk of cancer-related complications such as pathological fractures of the appendicular skeleton including the femur. Primaries from paired structures such as the breast, prostate, lungs, thyroid and kidneys are known to metastasise to bone commonly, with the femur being the most likely long bone to be affected by bony metastasis (44%) [1]. Patients with metastatic pathological femur fractures are associated with higher mortality rates of 60–83% at 1-year and 70–94% at 2 years after surgical treatment [2–9] compared with the 1-year mortality rate of about 12 to 37% for traumatic hip fractures [10]. Nevertheless, surgical intervention may be necessary to restore function and provide pain relief. Many of these patients are at the terminal stage of the disease and any treatment would be at best palliative.

Orthopaedic surgeons are faced with a difficult choice regarding the palliative value of surgical intervention for pathological femoral fractures. These patients are often medically compromised and have uncertain prognosis. There is also a lack of evidence-based data to guide treatment strategy and results are uncertain [11]. Nevertheless, life expectancy is one of the most important factors amongst orthopaedic surgeons when deciding whether to operate on patients with femoral metastases and what method of surgical intervention to utilise if the decision to operate is made [11].

Goal of palliative treatment is to maximise patients’ welfare and quality of life in their remaining days and accurate assessment of their survival is needed to prevent unnecessary invasive procedures which may not be beneficial to patients [12]. Some risk factors for mortality have been identified including site of primary tumour [2–8] and preoperative functional status [2, 6–8] in the literature. However, there is no strong consensus on the other risk factors for survival in patients with metastatic pathological femur fractures. Many studies have tried to predict survival and identify risk factors in patients with skeletal metastases including upper and lower limb metastases [2, 4, 6–8], but few look at femoral fractures in isolation [5, 9]. Other studies only limited their scope to a particular type of surgery i.e. arthroplasty [4, 7] or intramedullary nail [9].

Estimation of survival by physicians is frequently inaccurate and prognostic tools have been shown to lead to more accurate estimation. This would help aid decision-making by surgeons and patients especially for patients with mid-term estimations [12]. The most recent and comprehensive are the updated models of Katagiri et al. [13] and model by Forsberg et al. [3] However, only the model by Forsberg et al. has been validated in several small external cohorts and a recent survey shows that utilisation of these models could be improved [12].

Nevertheless, this study hopes to contribute to this body of literature by studying trends and risk factors for survival in our relatively large patient cohort in our institution who has received surgical treatment for metastatic pathological fractures, and by doing so better inform surgical decision-making.

## Methods

This is a retrospective study of prospectively collected data in our hospital surgical registry. Patients who sustained and were surgically treated for metastatic pathological femur fractures between 2007 and 2014 were identified. Only patients with histological proof of metastases as well as available mortality data at 2 years were subsequently included. Patients with primary bone tumours and patients with known cancer with negative histology for malignancy were excluded from this study. Decision to operate on these patients was made by a multidisciplinary team comprising oncologists, orthopaedic surgeons and musculoskeletal radiologists.

Patients’ mortality data and clinical information were then retrieved from electronic patient records. Factors evaluated included age of surgery, American Society of Anaesthesiologists Physical Status score (ASA), Charlson comorbidity index score (CCI), laboratory data including preoperative serum albumin and haemoglobin values, site of primary tumour, presence of visceral or spinal metastases, time from diagnosis of cancer to occurrence of metastatic pathological fracture, type of surgical procedure performed, site of fracture and whether treatment was received for an actual or impending fracture.

Age, time of cancer diagnosis to treatment of pathological fracture and laboratory data such as serum albumin and serum haemoglobin based on routine preoperative assessment for surgery were treated as continuous variables. ASA scores were divided into two groups: ASA 1 and 2 and ASA 3 and 4. For CCI, as all patients had malignancy, weights were not assigned to the cancer diagnosis, and the scores were also divided into two groups (CCI of 0 and CCI ≥ 1). Oncologic diagnosis was divided into five groups—the top 4 primary cancers in our population (lung, breast, prostate and renal) and other sites. Presence of visceral metastases (lung, liver, adrenals and kidney and other organs) and spinal metastases were confirmed by computed tomography scan of the thorax, abdomen and pelvis or by nuclear bone scan imaging. All scans were reviewed by a consultant radiologist and the consultant orthopaedic surgeon. The type (actual or impending) and location of fracture (divided into neck of femur, intertrochanteric/subtrochanteric and shaft/distal femur) was confirmed by plain radiograph. Surgeries performed included joint replacement with bipolar hemiarthroplasty and fixation with intramedullary nail or plate and screws.

## Statistical analysis

Univariate analysis logistic regression using Cox proportional-hazards model was performed on the above variables to determine if they were risk factors for mortality after treatment for pathological femur fractures. Statistical significance was defined as a *P* value of ≤ 0.05. The various variables that were found to be significant factors after univariate analysis (*P* < 0.05) for survival were entered into a Cox regression model with a backward stepwise approach. Remaining variables were considered to be significant risk factors if their odds ratio was significant at *P* < 0.05 after multiple regression analysis. All statistical analyses were performed using SPSS, version 21 (SPSS Inc., Chicago, IL).

## Results

One hundred fourteen patients who underwent treatment for metastatic pathological fractures in our institution were identified and only 112 of these patients had available 2-year follow-up with 117 femurs operated on. Two patients were excluded as their mortality data were unavailable. The patient demographics for this study are presented in Table 1.

**Table 1:** Patient demographics

Description		Details	
No. of patients (male:female)		112 (45:67)	
No. of femurs		117 (5 bilateral cases)	
Age of surgery		65.5 (54.3–71.8)	
ASA status (1 and 2:3 and 4)		51:61	
CCI (0:≥ 1)		71:41	
Albumin		32 (28–36)	
Haemoglobin (Hb)		11.5 (10.2–12.9)	
Site of primary tumour	Breast	33	(30%)
	Prostate	10	(9%)
	Lung	29	(26%)
	Renal	10	(9%)
	Others	30	(27%)
Presence of visceral metastases (yes:no)		65:47	
Presence of spinal metastases (yes:no)		82:30	
Time of diagnosis of cancer to incidence of pathological fracture (months)		14 (2–48)	
Pathology (Actual fracture:impending fracture)		61:51	
Site of fracture/lesion	Neck of femur	37	
	Intertrochanteric	20	
	Subtrochanteric	27	
	Shaft/distal femur	33	
Type of surgical procedure	Bipolar hemiarthroplasty	27	
	Intramedullary nail	79	
	Plate and screws	11	

Median values and interquartile ranges reported for age, albumin, haemoglobin and time of diagnosis of cancer to incidence of pathological fracture

Median survival for the whole study was 185 days (range 5–> 730 days, the lower and upper quartiles are 60 and 554 days respectively), while Kaplan-Meier mortality estimates were 50%, 67% and 86% at 6 months, 1 year and 2 years respectively.

### Univariate analysis of risk factors

Univariate analysis identified serum albumin, serum haemoglobin, site of primary tumour, presence of visceral metastases and time from diagnosis of cancer to incidence of pathological fracture as risk factors for survival after treatment (Table 2).

**Table 2:** Univariate analysis of risk factors of survival using Cox regression

Variable	Hazard ratio	95% CI	***P*** value	Reference/remarks
Age of surgery	1.008	0.992–1.024	0.333	For every year increase in age
ASA status (2:3)	1.506	1.004–2.257	0.480	ASA 3 against ASA 2
CCI (0:≥1)	1.241	0.822–1.873	0.305	No comorbidities against CCI ≥ 1
Albumin	1.100	1.062–1.139	< 0.001	For every 1 g/L decrease
Haemoglobin (Hb)	1.159	1.028–1.307	0.016	For every 1 g/dL decrease
Site of primary tumour				Lung as reference
Breast	0.394	0.223–0.697	0.001	
Prostate	0.426	0.192–0.945	0.036	
Renal	0.758	0.356–1.615	0.473	
Others	1.128	0.667–1.907	0.653	
Presence of visceral metastases (yes:no)	1.675	1.110–2.535	0.014	Visceral metastasis present against absent
Presence of spinal metastases (yes:no)	1.071	0.678–1.689	0.769	Spinal metastasis present against absent
Time from diagnosis of cancer to incidence of pathological fracture	0.995	0.992–0.999	0.016	For every month between diagnosis of cancer to fracture
Pathology (actual fracture:impending fracture)	1.185	0.791–1.774	0.411	Treatment of actual fracture against treatment of impending fracture
Site of fracture/lesion				Shaft/distal femur as reference
Neck of femur	0.722	0.427–1.221	0.224	
Intertrochanteric/subtrochanteric	0.833	0.511–1.360	0.465	5 bilateral cases excluded
Type of surgical procedure (bipolar hemiarthroplasty:intramedullary nail)	0.854	0.521–1.401	0.533	Bipolar against nail 15 cases excluded (10 plate and screws and 5 bilateral)

Age, gender, ASA, CCI, serum haemoglobin levels, presence of spinal metastases, whether the fracture was actual or impending, site of fracture/lesion and type of surgical procedure performed were not significant risk factors for survival.

### Multivariate analysis of risk factors

**Fig. 1:**
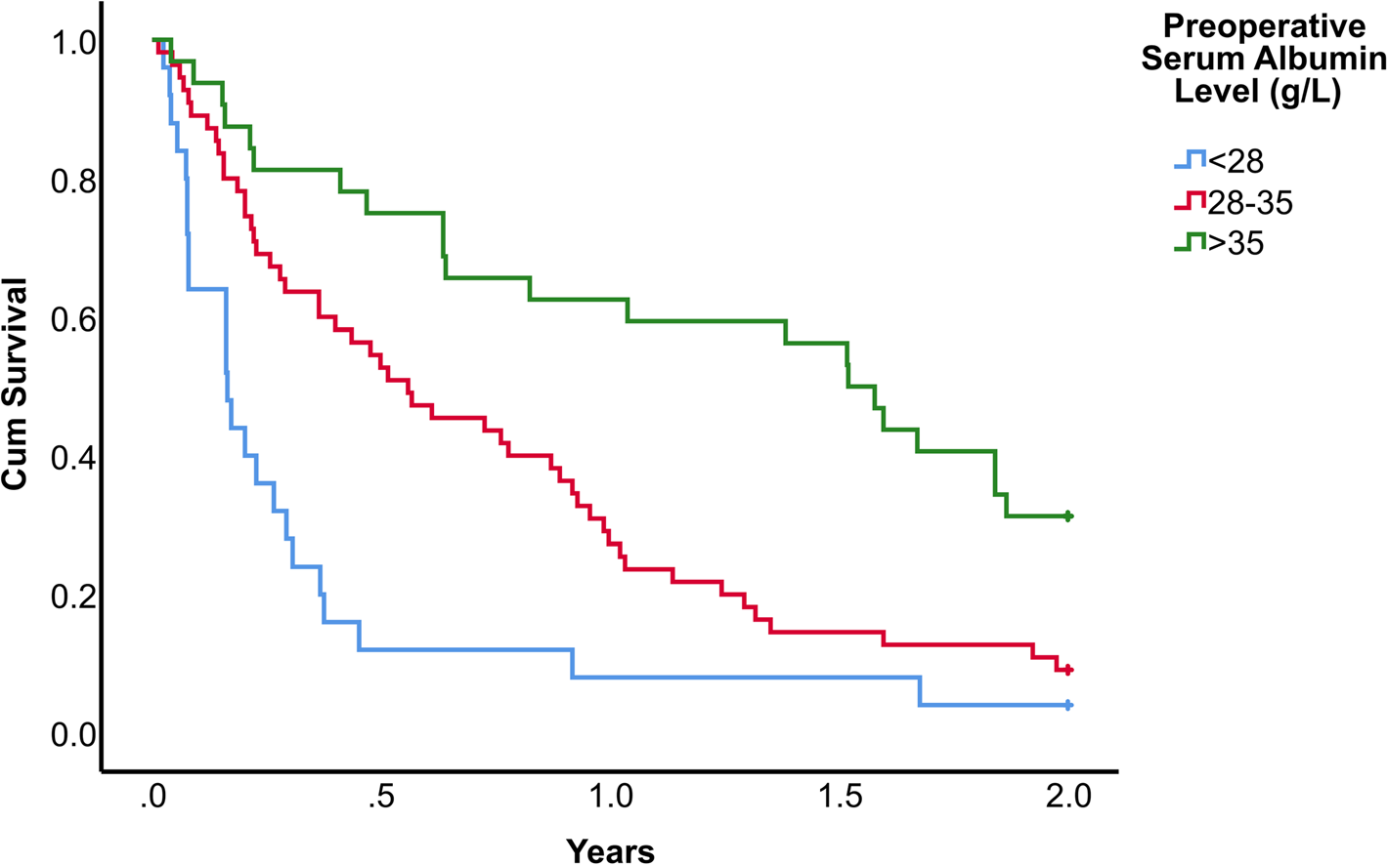
Kaplan-Meier survival estimate according to the degree of hypoalbuminemia

**Fig. 2:**
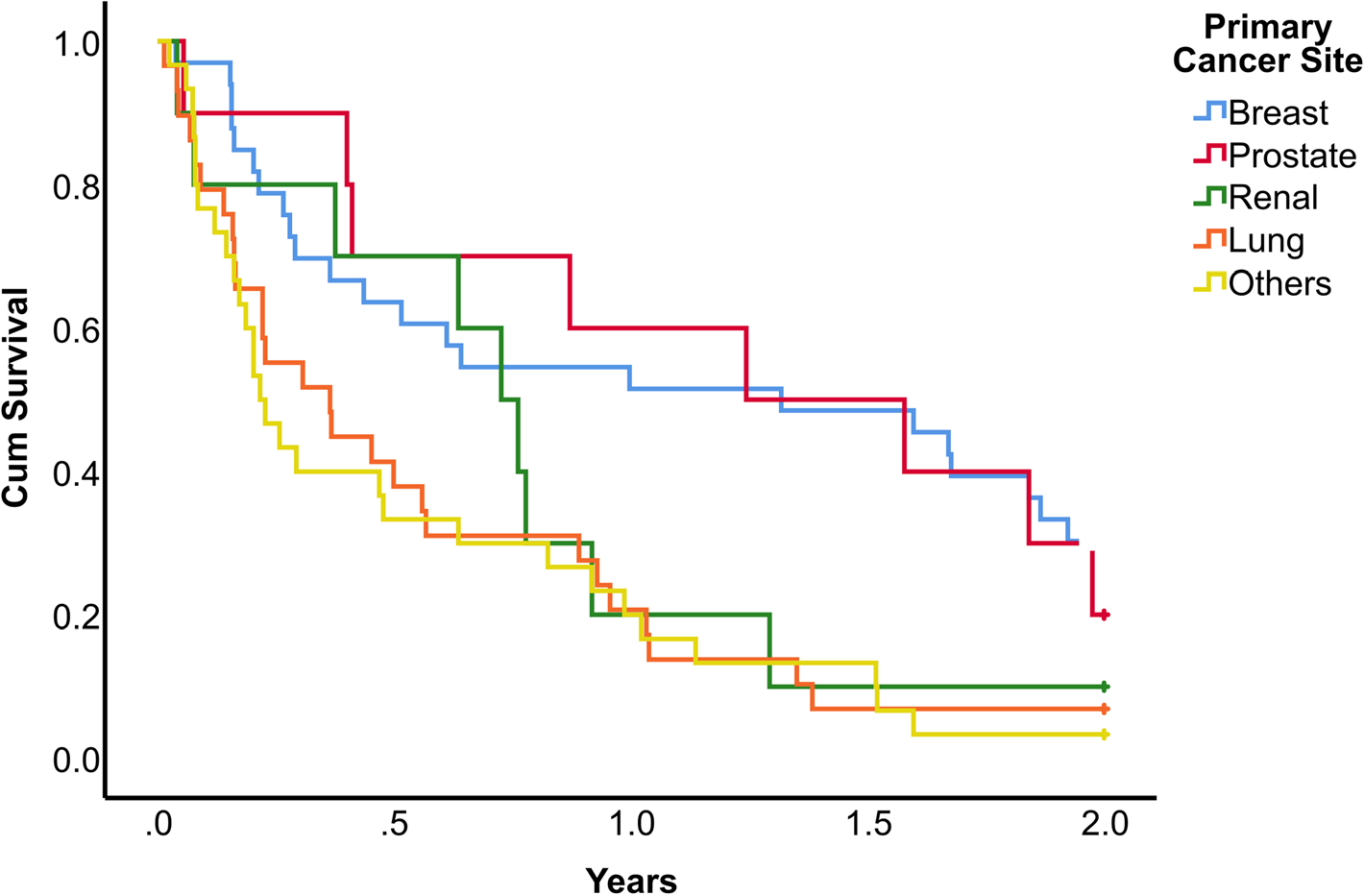
Kaplan-Meier survival estimate according to the primary cancer site

However, Cox regression analysis of risk factors revealed that preoperative serum albumin (hazard ratio for every 1 g/L decrease in albumin = 1.096, 95% CI [1.0.58–1.137], *P* < 0.001) and site of primary tumour (hazard ratio of breast cancer over lung cancer = 0.468, 95% CI [0.264–0.829], *P* < 0.009) were independent risk factors for survival (Table 3). Figure 1 shows the Kaplan-Meier survival estimate of our study group stratified according to the degree of hypoalbuminemia, while Fig. 2 shows the Kaplan-Meier survival estimate according to the primary cancer site. One-year survival for patients with albumin > 35, 28–35 and < 28 as stratified using Child-Pugh score for liver cirrhosis were 63%, 27% and 8% respectively (Fig. 1). One-year survival for patients with breast, prostate, renal, lung and all other patients were 52%, 60%, 20%, 21%, 20% respectively (Fig. 2).

**Table 3:** Cox regression of multiple risk factors for mortality after treatment for pathological hip fractures

Variable	Hazard ratio	95% CI	*P* value	Reference
Serum albumin	1.096	1.058–1.137	< 0.001	For every 1 g/L decrease
Primary cancer				Lung cancer
Breast	0.468	0.264–0.829	0.009	
Prostate	0.540	0.242–1.207	0.133	
Renal	0.770	0.361–1.641	0.499	
Others	1.389	0.841–2.368	0.228

### Other findings

Out of all the risk factors in our study, only the presence of visceral metastases strongly correlates with albumin (*P* = 0.009) (Table 4).

**Table 4:** Comparison of albumin levels in patients with and without visceral metastases

Presence of visceral metastases	Number	Mean albumin (g/L)	Mean difference	95% CI	***P*** value
NO	47	33.51 ± 4.52	2.76 ± 1.03	0.71–4.80	0.009
YES	65	30.75 ± 6.42			

## Discussion

Mortality rates in this study group are consistent with literature. We report a 30-day mortality of 13.4% which is similar to that previously reported (2.6 to 12%) [14–16]. One-year and 2-year mortality in our patients were 67% and 85.7% respectively, compared to previously reported mortality rates of 60–83% at 1 year and 70–94% at 2 years [2–9].

Regarding short-term outcomes, poor ASA and functional status have been previously found to be significant risk factors for 30-day mortality [15]*.* Previous studies observed that patients who were underweight [14], had rapid-growth tumours, visceral metastases, internal fixation or no postoperative chemotherapy [15] have higher mortality at 30 days but results were not statistically significant. On the other hand, age, gender, blood loss, blood transfusion, duration of surgery, primary cancer type, major bony resection and CCI were not found to be related to survival [14–16].

Many studies had also investigated risk factors for longer-term survival. However, Sorenson et al. found that predictors of survival were inconsistent amongst previous studies and cited that the reason might be because these studies on patients undergoing surgical treatment for metastatic lesions exhibit a great extent of heterogeneity [7]. Furthermore, different primary cancers contribute unequally to the various study groups and other studies include metastatic lesions at other sites including shoulder and the spine with differing proportions.

The common risk factor of survival across all studies is the site of the primary cancer leading to the pathological fracture. The link between primary site of cancer and long-term survival in patients with pathological femur fracture has been well-established [2–8]. Multivariate analysis of the risk factors for survival in this study demonstrates that patients with breast cancer have significantly better prognosis than patients with lung cancer.

Other factors previously studied and found to be significant in at least one study include preoperative serum haemoglobin, presence of visceral metastases, presence of spinal metastases, presence of brain metastases, number of bony metastases, whether the fracture was an actual or impending fracture, duration from time of cancer diagnosis to incidence of pathological fracture, type of procedure performed, functional status, presence of adjuvant therapy and ASA status [2–8].

In this study, risk factors that were significant in univariate analysis but ultimately not found to be independent risk factors after multivariate analysis include serum haemoglobin, presence of visceral metastases and time from diagnosis of cancer to incidence of pathological fracture as risk factors for survival after treatment.

Apart from site of primary cancer, serum albumin is the other independent risk factor for mortality in this study. Serum albumin is a readily available parameter to evaluate patient’s nutritional status [17] and provides useful prognostic significance in cancer survival [18] and traumatic hip fractures [19–21]. Preoperative serum albumin may be an indicator of patient’s ability to withstand surgical impact and early rehabilitation. It has also been shown to be useful for reducing complications in orthopaedic patients by allowing us to screen and treat those at risk [17]. Advantages of using serum albumin as a prognostic tool include the fact that it is a simple, inexpensive, and reproducible laboratory marker. Normal albumin levels have been found to correlate with higher survival in metastatic lesions at other body sites, such as the pelvis [22]. Few studies however have studied the role of albumin as a prognostic factor in pathological femoral fractures.

Nathan et al. looked into the effect of albumin on survival after treatment of pathological femur fractures and found albumin significant only in univariate analysis and not after multivariate regression [2]. Katagiri et al. noted that even though laboratory data including albumin are known to be prognostic factors for some malignancies, they have not been sufficiently investigated as prognostic factors in the past [13]. Katagiri et al. demonstrated that laboratory data can be a significant prognostic factor and included albumin in their prediction model—patients with albumin < 37 g/L had poorer prognosis in their model [13]. This prognostic model was based largely on non-surgically treated patients and may not be applicable to potential surgical candidates [23]. Patients who present with an actual fracture or impending fracture requiring surgery are at a later stage of their malignancy and this may explain why the average serum albumin level in our study group with only surgical-treated patients is 32 g/L. Cox regression analysis in our study demonstrates worse prognosis for every 1 g/L decrease in albumin. This suggests that prognostic models may benefit from looking at the adverse effect that even lower levels of serum albumin have on prognosis; however, further studies are needed to fully validate this.

Serum albumin may be a useful prognostic factor for survival and commonly utilised as an indicator for malnutrition. Nevertheless, there is limited evidence that nutritional supplementation may improve prognosis in patients with low serum albumin levels. Gupta et al. suggested that since low levels of serum albumin are linked with poorer outcome in cancer patients, serum albumin can perhaps be utilised as an independent indicator of the need for nutrition intervention [18]. Nevertheless, the study noted the absence of clinical trials demonstrating that raising albumin levels, by means of intravenous infusion or hyperalimentation, decreases the excess risk of mortality in cancer patients, and by extension, patients with metastatic femoral fractures. A Cochrane review also found minimal evidence to suggest that nutritional supplementation can reduce mortality in patients with hip fractures [24].

This study found a significant correlation between albumin and presence of visceral metastases. This finding needs to be validated by further studies. If so, serum albumin may be interpreted as an indicator of general physiological well-being, and its potential for optimization be limited by the presence of visceral metastasis.

This study shows that there could be a role for albumin in prognostication of survival in patients with metastatic femur fractures. There may be value in measuring serum albumin routinely on admission to aid decision-making with regard to surgical treatment. Prudence is imperative before commencing surgical treatment on patients who have hypoalbuminaemia or are extremely cachectic and malnourished as they may not benefit from palliative surgery. Further research is required to determine if clinical scoring of prognosis to guide treatment in patients with metastatic pathological femur fractures will improve outcomes.

### Limitations

This was a single-centre retrospective study using prospectively collected data with all the limitations inherent to such design.

This study did not include patients who were treated non-surgically as records were only obtained from our hospital surgical registry. This study was unable to capture date on patient who underwent adjuvant treatment modalities including chemotherapy, hormonal therapy and radiotherapy, which may affect their survival.

Data on the peri-operative functional outcomes which had been previously found to be significant risk factors for survival was not included in this study [2, 6, 7].

### Strengths

This study represents a more homogenous cohort of pathological femur fractures compared to previous studies.

## Conclusion

Preoperative serum albumin level and primary tumour site are independent prognostic factors of survival in patients treated for pathological femur fractures. Patients with breast cancer have a better prognosis compared to lung cancer patients. However, studies with higher number of patients are required to verify the prognostic role of albumin. Surgical treatment of metastatic femoral fractures in patients with hypoalbuminaemia should proceed with caution. Peri-op nutritional assessment and survival prognostication are pertinent for this subset of patients to guide treatment and affect clinical outcomes. Further clinical trials will be needed to ascertain if nutritional supplementation may be of benefit to patients with low albumin.

## Data Availability

The datasets used and/or analysed during the current study are available from the corresponding author on reasonable request.

## Abbreviations

ANA: American Society of Anaesthesiologists
CCI: Charlson comorbidity index

## References

[1] Narazaki DK, de Alverga Neto CC, Baptista AM, Caiero MT, de Camargo OP (2006). Prognostic factors in pathologic fractures secondary to metastatic tumors. Clinics..

[2] Nathan SS, Healey JH, Mellano D, Hoang B, Lewis I, Morris CD (2005). Survival in patients operated on for pathologic fracture: implications for end-of-life orthopedic care. J Clin Oncol..

[3] Forsberg JA, Wedin R, Boland PJ, Healey JH (2017). Can we estimate short- and intermediate-term survival in patients undergoing surgery for metastatic bone disease?. Clin Orthop Relat Res..

[4] Schneiderbauer MM, von Knoch M, Schleck CD, Harmsen WS, Sim FH, Scully SP (2004). Patient survival after hip arthroplasty for metastatic disease of the hip. J Bone Joint Surg Am..

[5] Sarahrudi K, Greitbauer M, Platzer P, Hausmann J-T, Heinz T, Vécsei V (2009). Surgical treatment of metastatic fractures of the femur: a retrospective analysis of 142 patients. J Trauma..

[6] Hill T, D’Alessandro P, Murray K, Yates P (2015). Prognostic factors following pathological fractures. ANZ J Surg..

[7] Sørensen MS, Gerds TA, Hindsø K, Petersen MM (2016). Prediction of survival after surgery due to skeletal metastases in the extremities. Bone Joint J..

[8] Hansen BH, Keller J, Laitinen M, Berg P, Skjeldal S, Trovik C (2004). The Scandinavian Sarcoma Group skeletal metastasis register survival after surgery for bone metastases in the pelvis and extremities. Acta Orthop Scand.

[9] Piccioli A, Rossi B, Scaramuzzo L, Spinelli MS, Yang Z, Maccauro G (2014). Intramedullary nailing for treatment of pathologic femoral fractures due to metastases. Injury.

[10] Foster KW. Hip fractures in adults. UpToDate. 2017 Oct.

[11] Araki N, Chuman H, Matsunobu T, Tanaka K, Katagiri H, Kunisada T, et al. Factors associated with the decision of operative procedure for proximal femoral bone metastasis: questionnaire survey to institutions participating the Bone and Soft Tissue Tumor Study Group of the Japan Clinical Oncology Group. J Orthop Sci. 22(5):938–45. 10.1016/j.jos.2017.05.012 28629828

[12] Willeumier JJ, van de Sande MAJ, van der Wal RJP, Dijkstra PDS (2018). Trends in the surgical treatment of pathological fractures of the long bones. Bone Joint J..

[13] Katagiri H, Okada R, Takagi T, Takahashi M, Murata H, Harada H (2014). New prognostic factors and scoring system for patients with skeletal metastasis. Cancer Med..

[14] Kreul SM, Sorger JI, Rajamanickam VP, Heiner JP (2016). Updated outcomes of prophylactic femoral fixation. Orthopedics..

[15] Tsuda Y, Yasunaga H, Horiguchi H, Fushimi K, Kawano H, Tanaka S (2016). Complications and postoperative mortality rate after surgery for pathological femur fracture related to bone metastasis: analysis of a nationwide database. Ann Surg Oncol..

[16] Sørensen MS, Hindsø K, Hovgaard TB, Petersen MM (2016). Extent of surgery does not influence 30-day mortality in surgery for metastatic bone disease: an observational study of a historical cohort. Hanaoka. K, ed. Medicine.

[17] Cross MB, Yi PH, Thomas CF, Garcia J, Della Valle CJ (2014). Evaluation of malnutrition in orthopaedic surgery. J Am Acad Orthop Surg..

[18] Gupta D, Lis CG (2010). Pretreatment serum albumin as a predictor of cancer survival: a systematic review of the epidemiological literature. Nutr J..

[19] Kumar V, Alva A, Akkena S, Jones M, Murphy PN, Clough T (2014). Are albumin and total lymphocyte count significant and reliable predictors of mortality in fractured neck of femur patients?. Eur J Orthop Surg Traumatol..

[20] Kieffer W, Rennie C, Gandhe A (2013). Preoperative albumin as a predictor of one-year mortality in patients with fractured neck of femur. Annals of The Royal College of Surgeons of England..

[21] Laulund AS, Lauritzen JB, Duus BR, Mosfeldt M, Jørgensen HL (2012). Routine blood tests as predictors of mortality in hip fracture patients. Injury..

[22] Krishnan CK, Han I, Kim H-S (2017). Outcome after surgery for metastases to the pelvic bone: a single institutional experience. Clin Orthop Surg..

[23] Cassidy JT, Baker JF, Lenehan B (2018). The role of prognostic scoring systems in assessing surgical candidacy for patients with vertebral metastasis: a narrative review. Global Spine J..

[24] Avenell A, Curtain JP, Mak JC, Myint PK, Smith TO (2016). Nutritional supplementation for hip fracture aftercare in older people. Cochrane Database Syst Rev..

